# Fused Deposition Modeling of Isotactic Polypropylene/Graphene Nanoplatelets Composites: Achieving Enhanced Thermal Conductivity through Filler Orientation

**DOI:** 10.3390/polym16060772

**Published:** 2024-03-11

**Authors:** Zhongzui Wang, Qinjie Yang, Xinmei Zheng, Shuai Zhang, Pan He, Rui Han, Gang Chen

**Affiliations:** 1 School of Materials Science and Engineering, Xihua University, Chengdu 610039, China; 2 Engineering Research Center of Intelligent Air-Ground Integration Vehicle and Control, Xihua University, Ministry of Education, Chengdu 610039, China; hepan-2009@163.com; 3 Sichuan Provincial Engineering Research Center of Functional Development and Application of High-Performance Special Textile Materials, Chengdu Textile College, Chengdu 611731, China

**Keywords:** 3D printing, fused deposition modeling, isotactic polypropylene, graphene nanoplatelets, thermal conductivity, alignment

## Abstract

High-performance thermally conductive composites are increasingly vital due to the accelerated advancements in communication and electronics, driving the demand for efficient thermal management in electronic packaging, light-emitting diodes (LEDs), and energy storage applications. Controlling the orderly arrangement of fillers within a polymer matrix is acknowledged as an essential strategy for developing thermal conductive composites. In this study, isotactic polypropylene/GNP (iPP/GNP) composite filament tailored for fused deposition modeling (FDM) was achieved by combining ball milling with melt extrusion processing. The rheological properties of the composites were thoroughly studied. The shear field and pressure field distributions during the FDM extrusion process were simulated and examined using Polyflow, focusing on the influence of the 3D printing processing flow field on the orientation of GNP within the iPP matrix. Exploiting the unique capabilities of FDM and through strategic printing path design, thermally conductive composites with GNPs oriented in the through-plane direction were 3D printed. At a GNP content of 5 wt%, the as-printed sample demonstrated a thermal conductivity of 0.64 W/m · K, which was 1.5 times the in-plane thermal conductivity for 0.42 W/m · K and triple pure iPP for 0.22 W/m · K. Effective medium theory (EMT) model fitting results indicated a significantly reduced interface thermal resistance in the through-plane direction compared to the in-plane direction. This work shed brilliant light on developing PP-based thermal conductive composites with arbitrarily-customized structures.

## 1. Introduction

As electronic devices are developing toward miniaturization and integration, managing heat dissipation has emerged as a critical concern [1,2]. Effective thermal management is essential for maintaining device performance stability, extending its service life, and enhancing energy efficiency. Generally, thermal management materials are characterized by their high thermal conductivity, excellent electrical insulation, and good processability [3]. Polymer composites are candidate materials in the field of thermal management due to their excellent comprehensive properties, such as lightweight, mild processing conditions, and resistance to chemical corrosion [4]. However, the inherently low thermal conductivity of most polymers, typically ranging from 0.1 to 0.5 W/(m · K), restricts their broader adoption as thermally conductive materials in the industry to a certain extent [4,5].

Introducing high thermal conductive fillers into polymers to prepare filled thermally conductive composites has been considered a straightforward approach to preparing thermally conductive materials [6]. Especially when the filler is oriented and well distributed in the matrix, the contact thermal resistance among fillers and the interface thermal resistance between the filler and the polymer matrix decrease, enabling the achievement of high thermal conductivity at lower filler concentrations [7,8,9]. Moreover, minimized filler loading helps reduce defects and prevents the degradation of mechanical properties. Therefore, directing the preferential alignment of fillers within the matrix has emerged as an effective strategy for optimizing thermal conductivity [3,10]. Two-dimensional sheet materials such as graphene and hexagonal boron nitride are widely used in the development of thermal conductive composites. Notably, their in-plane thermal conductivity is significantly higher than in the through-thickness direction, exhibiting obvious thermal anisotropy [11,12]. This is due to the in-plane lattice vibration causing minimal phonon scattering during heat conduction, while inter-layer propagation encounters greater resistance [13,14]. To fully harness the ultra-high thermal conductivity of two-dimensional materials in-plane, researchers have combined orientation control strategies with techniques such as tape casting [15], stretching [16,17,18], ice crystal growth [19,20], etc., to prepare a series of thermal conductive composites [21].

In some thermal management fields, particularly for applications demanding complex shapes and structures such as LED skeletons and heat sinks, there is a mushroom growing for thermally conductive devices with customizable features [22,23]. To this end, in recent years, 3D printing has been employed to prepare thermally conductive composite products. As an extrusion 3D printing technology, fused deposition modeling (FDM) relies on the principle where the thermoplastic filament is fed into a liquefier by a pair of driving gears and heated. Upon extruded from the nozzle, the 3D part is then deposited layer-by-layer following the slice path of the STL format. FDM stands out by its cost-effectiveness, convenience, mild processing conditions, and broadening suitable materials, making it one of the most widely used 3D printing technologies. Recently, various printable materials and equipment have been developed to adapt to the trend of FDM from primary prototyping to high performance and functionality [24,25,26,27,28]. Based on the layer-by-layer stacking principle, FDM has been more widely employed for the fabrication of thermally conductive composites. FDM processing is similar to traditional extrusion molding to a certain extent. Based on layer-by-layer manufacturing principles, fused deposition modeling (FDM) can create advanced geometries and has been started to be used more frequently for the fabrication of thermally conductive composites. It has been well accepted that FDM involves strong flow fields such as shear fields and tensile fields, which helps to achieve precise control of filler orientation and distribution state from micro to macro levels [29,30,31,32]. For example, Jia et al. used FDM to fabricate graphite/polyamide six composites, achieving an orientation of flake graphite along the through-plane direction by regulating the building direction [33]. Remarkably, with the graphite content at 50 wt%, the thermal conductivity of the printed sample reached 5.5 W/m · K, significantly exceeding the 2.4 W/m · K of the injection molded sample. Since micron-level flake graphite possesses weak intermolecular forces, its alignment along the printing direction during the FDM process is facilitated. Conversely, due to their large specific surface area, nanoscale fillers tend to agglomerate during processing, making their alignment difficult. To this end, Jing et al. used solid-state shear milling to achieve in-situ exfoliation and uniform dispersion of graphene nanoplatelets (GNPs) in the LLDPE matrix [34]. Subsequently, FDM was used to achieve precise orientation and arrangement of GNPs within the printed parts. The interfacial thermal resistance of the composites was also improved due to the compatibilization effect of solid-state shear milling. Consequentially, the printed parts obtained a thermal conductivity of 3.43 W/m · K. Furthermore, Guo et al. used FDM to fabricate graphene/TPU composites, achieving a high thermal conductivity of 12 W/m · K with a 45 wt% graphene content [35]. Interestingly, due to the high graphene content, the deposited filament exhibited distinctive vortex orientation morphologies after FDM printing, with an orientation degree of approximately 0.30–0.45. Such printed parts demonstrated considerable promise for the thermal management of lithium batteries. The maximum temperature of the battery was reduced by about 5.7 °C compared to the pure TPU shell.

Here, we noticed that polypropylene (PP) thermally conductive composite materials are frequently required in applications such as LED lamps and substrates due to their superior chemical resistance and thermal stability. However, the inherent high regularity of the PP molecular chain facilitates crystallization during FDM processing, leading to shrinkage and warping [36]. This often results in the printed part detaching from the build platform, eventually causing printing failures. After adding fillers such as graphene, it may also act as a heterogeneous nucleating agent to accelerate the crystallization kinetics of PP [37]. Therefore, there are currently few studies on 3D printing of PP and its based composites. Additionally, the incorporation of fillers such as graphene can serve as heterogeneous nucleating agents, further accelerating the crystallization kinetics of PP. These factors make research on the 3D printing of polypropylene and its composites complex and challenging. To the best of our knowledge, there are currently few studies on the 3D printing of PP-based composites. Yuval Shmueli initially implemented FDM printing of iPP/GNP composites, indicating that the inclusion of GNP facilitated the templating of the molecular chains on the surface of GNP but also hindered the formation of the shish-kebab structure during the FDM process [38]. Note that this study utilized a lower molecular weight (Mw) iPP, and the examination of the impact of GNP orientation on thermal conductivity appears somewhat limited. Furthermore, high concentrations of GNP could potentially compromise the insulating properties of the composites. In the present study, we first used ball milling to achieve a uniform mixture of GNP and PP, and then we further combined this with melt extrusion processing to prepare iPP/GNP composite filaments. To ensure that the thermal conductivity of printed parts was improved without affecting the electrical insulation of the material, the graphene content was below 5 wt%. Furthermore, the building direction was meticulously designed, allowing for the manipulation of GNPs to orient along the chosen direction subjected to the flow field of FDM processing. With a GNP content of 5 wt%, the as-printed samples exhibited a thermal conductivity of 0.64 W/m · K. This study provides valuable insights for the development of thermal management systems tailored to the specific requirements of complex structures of PP-based thermally conductive composites, including applications such as LED lamps.

## 2. Materials and Methods

### 2.1. Materials

The isotactic polypropylene (iPP, trademarked as T03) was supplied by Sinopec Maoming Petrochemical Company, Guangdong, China. Graphene nanosheets (GNP, trademarked as XF022-1) were supplied by Nanjing Xianfeng Nano Co., Ltd., Nanjing, China, featuring a wafer diameter of 1–3 μm and a thickness of 1–5 nm.

### 2.2. Sample Preparation

**Figure 1:**
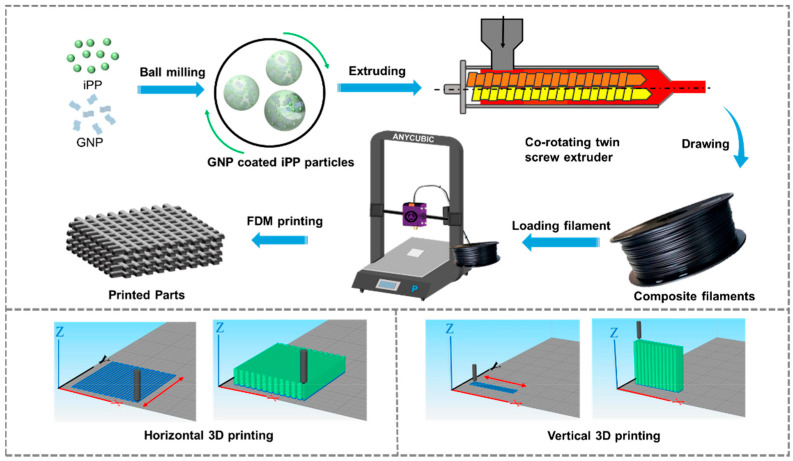
Schematic diagram of the fabrication of iPP/GNP composite filaments and their FDM process.

The schematic diagram of the preparation of iPP/GNP composites and its 3D printing process is shown in Figure 1. Specifically, to make GNPs evenly coated on the surface of iPP particles, the iPP and GNPs with various loadings of 1, 3, and 5 wt% were proportionally mixed and then subjected to planetary ball milling (QM-DY2, Nanjing Nanda Instrument Co., Ltd., Nanjing, China) for 120 min, to prepare iPP/GNP composite powder. The milling was carried out at a rotational speed of 400 rpm with a mass ratio of steel ball mass to composite powder of 10:1. After that, the composite powder was melt-extruded through a co-rotating twin-screw extruder (TSE-30A, Nanjing Ruiya Extrusion System Co., Ltd., Nanjing, China). The extrusion process was operated at a speed of 30 rpm, with temperature settings at 170 °C, 200 °C, and 200 °C for feeding section, melting section, and plasticizing section, respectively. The filament was drawn at a speed of 2.58 m/min and cooled in a circulating water bath to ensure proper cooling and solidification, eventually maintaining a diameter of 1.75 ± 0.05 mm. For comparative studies, neat iPP was processed under the same conditions. The 3D printing process was as follows: firstly, UG software 10.0 was used to construct the 3D solid models with a size of 14 × 14 × 4 mm^3^ and exported into STL format, and further imported to Simplify 3D software 4.0 for slicing. The corresponding slicing parameters were set in Table 1. During the entire printing process, the printer door is closed to minimize heat loss. The sliced model was exported into G-code format and transferred to the FDM equipment (3D-Hope F160, Chengdu Yuanhao 3D Technology Co., Ltd., Chengdu, China) for further 3D printing. For the further 3D printing. Two distinct construction strategies were employed to fabricate the parts and examine the impact of various orientations of GNP on the thermal conductivity of the as-printed samples. One was the FDM printing direction parallel to the length direction of the sample, defined as FP. Another was the FDM printing direction parallel to the thickness direction of the sample, which is defined as VP. Accordingly, eight different FDM printed samples were obtained: FP0, FP1, FP3, FP5, VP0, VP1, VP3, and VP5. The number in the sample name represented the GNP content. For example, VP5 denoted that the orientation of the printed part was aligned with its thickness direction, and the GNP content was 5 wt%.

### 2.3. Material Characterization

The rheological performance of iPP/GNP composites was carried out on a rotating rheometer (DHR-1, TA instrument, Newcastle, DE, USA) with parallel plate geometry (25 mm diameter). The test temperature was 200 °C with a strain of 1%. The frequency sweep ranged from 100 to 0.01 rad/s. The morphologies of the iPP/GNP printed samples were observed by a scanning electron microscope. Before testing, the test specimens were cryogenically fractured in liquid nitrogen and then sprayed with gold on the cross-section. Finally, the as-prepared specimens were observed by (SEM, SU8010, Hitachi, Tokyo, Japan) with an acceleration voltage of 5 KV. DSC 25 (TA Instruments, Newcastle, DE, USA) was used to study the melting behavior of the composites. The sample of about 5 mg cut from the FDM printed sample was first heated from 40 °C to 200 °C at a heating rate of 30 °C/min to minimize the impact of β/α crystalline transformation on the test results during the heating process. The crystallinity (Xc) of iPP/GNP components was calculated by formula $\chi_{c}=\frac{\Delta H_{m}}{\left[\left(1-\varphi \right)\Delta H^{0}\right]}\times 100\%$, where $\Delta H_{m}$ represented the melting enthalpy measured by DSC, φ represented the mass fraction of the component in the blended sample; $\Delta H^{0}$ the melting enthalpy of pure PP in a completely crystallized state, with a value of 207 J/g. The crystal structure of iPP/GNP composites was determined by a high-performance X-ray diffractometer using Cu Kα radiation (Ultima IV, Rigaku, Japan) with a voltage of 45 KV, a scanning speed of 0.01°/s, and a scanning range of 2θ from 10° to 30°. A tensile test was carried out on a universal mechanical testing machine (CMT6104, Shenzhen new Sansi Measurement Technology Co., Ltd., Shenzhen, China). A dumbbell-shaped spline was printed for the mechanical test with a filling rate of 100%, and the printing angle was set parallel to the X-axis (0°). The dimensions of the spline were 65 mm in length, 10 mm in width, and 2 mm in height. In accordance with ASTM-D638 [39], the mechanical properties of the specimen were evaluated at room temperature using a tensile speed of 10 mm/min. The test was repeated five times for each group of samples, and the average value of these tests was recorded. The thermal conductivity of the printed sample was measured using a thermal constant analyzer (Hot Disk TPS 2200, Hot Disk AB, Gothenburg, Sweden). For the in-plane and through-plane thermal conductivity tests, the samples were prepared with dimensions of 14 mm in length), 13 mm in width, and 4 mm in thickness. During the testing procedure, a probe was positioned between two samples for 10 s, with a test power of 20 mW. Three samples from each group were tested, and the average value was recorded. The thermal conductivity *λ*(*T*) was calculated by the equation: $\lambda \left(T\right)=\alpha (T)\rho (T)C_{P}(T)$, where ρ(T) is the density, $C_{P}(T)$ represents the specific heat and α(T) is the thermal diffusion coefficient. $C_{P}(T)$ was determined by using the Modulated DSC method, α(T) was measured with the thermal constant analyzer, and ρ(T) was obtained through the buoyancy method. A thermal failure analyzer (220RD PCBA, Shenzhen Youce Technology Co., Ltd., Shenzhen, China) was used to carry out in-plane and through-plane infrared thermal imaging of the printed parts. Samples with a size of 14 × 13 × 4 mm^3^ were placed on a heating platform, heated to 100 °C, and maintained at this temperature for 20 min. Subsequently, the samples were quickly transferred to a steel plate at ambient temperature for natural cooling. Throughout this process, the surface temperature changes were recorded by the AnalyzIR software V5.0 integrated with infrared thermography.

## 3. Results

### 3.1. Rheological Properties of iPP/GNP Composites

**Figure 2:**
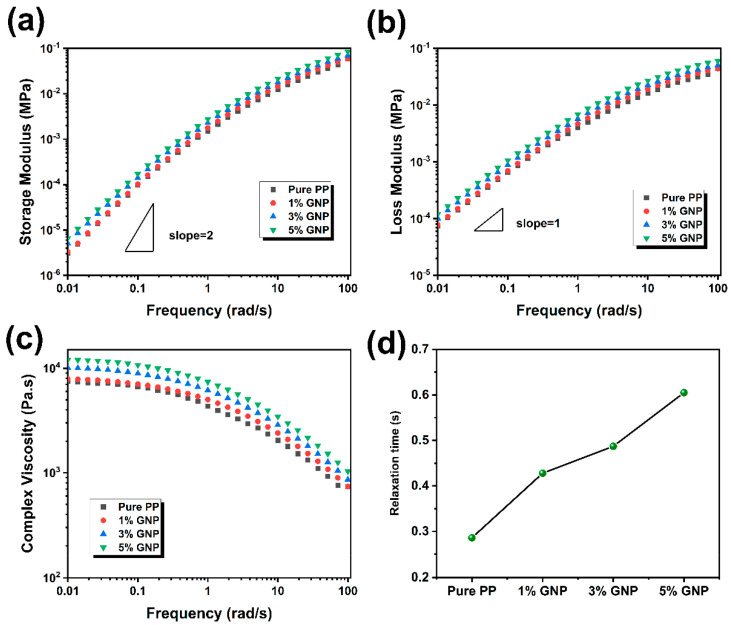
Rheological properties of iPP and iPP/GNP composites with various GNP contents: (**a**) Storage modulus, (**b**) Loss modulus, (**c**) Complex viscosity, and (**d**) Relaxation time.

An understanding of the rheological behavior of the material is crucial to the successful realization of the FDM process in terms of printability and quality of the printed parts. Accordingly, the rheological properties of iPP/GNP composites were studied. Figure 2a,b presents the frequency dependency of the storage (G′) and loss (G″) modulus of iPP/GNP composites as a function of GNP loading at 200 °C. The modulus of iPP was slightly enhanced within the entire frequency range with the addition of GNP. Moreover, incorporating GNP into iPP has been observed to enhance the G′ more than G″, suggesting the more pronounced effect of GNP on the elastic rather than the viscous response of the composites. Both the G′ and G″ modulus decreased as the frequency decreased, showing a typical liquid-like behavior due to the low loading of GNP. The relationship between complex viscosity and angular frequency is depicted in Figure 2c. According to the Cox-Merz rule, there is an approximate correspondence between the viscosity of the polymer under steady-state shear and the complex viscosity at the equivalent oscillation frequency, where the shear rate is equal to the oscillation frequency [40]. As can be seen, the complex viscosity of iPP/GNP composites decreased with the increasing angular frequency, exhibiting an obvious shear thinning behavior at approximately 1 s^−1^, thereby aiding in minimizing the risk of nozzle clogging and enhancing the flowability during the FDM extrusion process. The complex viscosity increased with the addition of GNPs across the entire frequency range. Furthermore, we calculated the relaxation time of the composites according to the Cox-Merz rule, as shown in Figure 2d. With the increasing content of GNP, the relaxation time of the molecular chain increased from 0.286 s for pure iPP to 0.605 s when the GNP was 5 wt%. The enhanced viscosity for the composites can be attributed to the restriction of polymer chain mobility due to the presence of GNPs. An elevated viscosity can present challenges in the FDM process, such as requiring higher extrusion pressures and potentially impacting the consistency and uniformity of extrusion, thus affecting print quality. However, research also indicates that the increased viscosity from adding nanoparticles can be advantageous [41]. This is primarily because a higher zero-shear viscosity during the deposition phase can help maintain the stability of the printed structure, thereby enhancing the accuracy of the printed parts. Moreover, the incorporation of nanoparticles can mitigate warping behaviors during iPP 3D printing to a certain extent. Noted that the complex viscosity of the composites with various GNP loadings was basically close at a high frequency of 100 rad/s, suggesting that the incorporation of GNPs on the viscoelastic behavior of the polymer matrix was almost negligible at a higher shear rate, likely due to the alignment of GNPs along the printing direction. To evaluate the shear applied to the filament, it was assumed that the extrusion of the filament passed through the nozzle was under a linear velocity gradient across the cross-section of the nozzle, maintaining a laminar flow state and with no slip at the nozzle wall. The stress in the nozzle can be estimated as $\sigma_{max}\approx \mu \frac{\nu }{h}=7340\;Pa\cdot s\times \frac{20\frac{mm}{s}}{0.2\;mm}\approx 0.73\;MPa$, according to the fundamental principles of fluid dynamics [38]. This value was almost two orders of magnitude higher than that observed at the onset of shear thinning, which was consistent with the orientation of GNPs.

### 3.2. Finite Element Simulation of FDM Extrusion Process for the Composites

**Figure 3:**
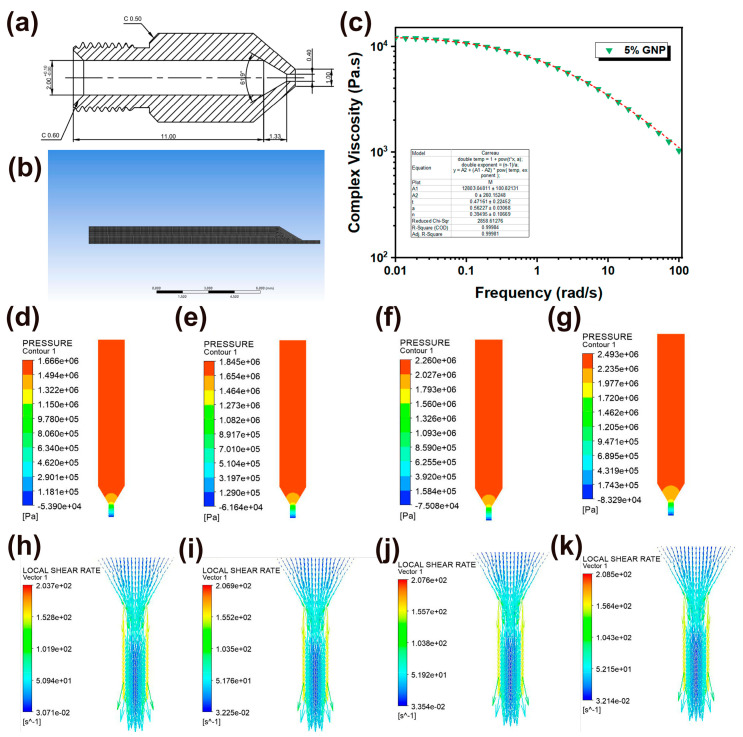
(**a**) cross-sectional view of the printing nozzle; (**b**) finite element meshing of two-dimensional nozzle model; (**c**) Carreau-Yasuda model for the parameters fitted to the iPP/GNP composites by taking 5 wt% GNP as an example; (**d**–**g**) Polyflow finite element simulation of pressure distribution within the cross-section of the nozzle for iPP, 1 wt% GNP, 3 wt% GNP, and 5 wt% GNP, respectively; (**h**–**k**) local shear rate distribution within the cross-section of the nozzle for iPP, 1 wt% GNP, 3 wt% GNP, and 5 wt% GNP, respectively.

Clarifying the distribution of pressure and shear fields during the 3D printing extrusion process is crucial for controlling the alignment behavior of GNPs, which is closely related to the thermal conductivity of the printed parts [42]. Accordingly, a Polyflow finite element was employed to simulate the melt flow behavior within the nozzle during the extrusion stage. Considering the central symmetry of the nozzle design, the melt flow state during the FDM extrusion process can be simplified as an analysis of the fluid flow on the cross-sectional. The detailed cross-sectional view and associated dimensions of the nozzle are shown in Figure 3a. The corresponding meshing is shown in Figure 3b. The element size of the mesh is 5 × 10^−2^ mm, generating 4823 elements. Since iPP/GNP composites were a non-Newtonian fluid and exhibited obvious shear-thinning behavior, the Carreau-Yasuda equation was used to model the rheological behavior of the composites due to its applicability in a wide shear range [43]. The Carreau-Yasuda equation is as follows:

$$\frac{\eta -\eta_{\infty }}{\eta_{0}-\eta_{\infty }}=\frac{1}{{\left[1+{\left(\lambda \dot{\gamma }\right)}^{a}\right]}^{\frac{1-n}{a}}}$$

where η is the viscosity corresponding to shear rate, $\eta_{0}$ is the zero-shear viscosity, and $\eta_{\infty }$ is ultimate shear viscosity. In addition, λ is the relaxation time, n is the power law exponent, and a is the constant controlling the viscosity transition rate from the zero-shear Newtonian plateau to the shear-thinning region. Taking the GNP content of 5 wt% as an example, the fitting resulting is shown in Figure 3c. Consequentially, the $\eta_{0}$ and $\eta_{\infty }$ were determined to be 1.28 × 10^4^ Pa·s  and 0 Pa·s, respectively. λ was 0.472 s, a was 0.562, and n was 0.395. It can be seen that the fitting degree was 100%, reflecting the reliability of the Carreau-Yasuda equation. The volume flow rate of the fluid at the of the flow channel was set to be 1.256 mm^3^/s, and it was also assumed that the flow velocity at the inner wall position of the nozzle was set to be zero (no-slip boundary condition) and the flow pattern was a laminar flow. Accordingly, the pressure field and shear field distribution results were obtained, as shown in Figure 3d–k. During the FDM extrusion process, the pressure was observed to be maximal at the inlet, gradually decreasing when entering the convergence area, and reaching a minimum at the outlet. This phenomenon occurred because, during the extrusion process, the volume flow rate was considered constant. The cross-sectional area of the flow channel at the inlet was large, and the corresponding speed was low, whereas the small cross-sectional area at the outlet corresponded to the maximum extrusion speed. According to Bernoulli’s equation, the pressure was greatest at the inlet and smallest at the outlet. Notably, the pressure drops gradually increased with the increase of GNP content, which could be attributed to the enhanced viscosity and flow resistance with the corporation of GNP, as demonstrated by the rheological results. This increased resistance to flow required higher pressures to extrude the material through the nozzle. During the 3D printing extrusion process, the filament initially traversed a long and broad channel with a 2 mm diameter upon entry into the liquefier flow channel. In this stage, the melt flow rate was relatively low, and the shear rate was almost negligible. Subsequently, the filament advanced into the convergence region, where the melt was simultaneously subjected to intense shear and extensional field, inducing the disordered GNPs to move progressively into an ordered structure. Finally, the flow channel narrowed further to 0.4 mm, suffering from the highest velocity and shear rate. However, the presence of a velocity gradient resulted in the highest flow velocity at the center of the nozzle, where the shear rate was zero; conversely, the flow velocity approached zero near the nozzle wall, where the shear rate reached its maximum. As shown in Figure 3h–k, with an increase in GNP content, there was a slight rise in the maximum shear rate experienced by the melt but maintained around 200 s^−1^. Eventually, GNPs rotated and aligned along the extrusion direction upon being subjected to the shear field.

### 3.3. Morphologies of FDM Printed iPP/GNP Samples

**Figure 4:**
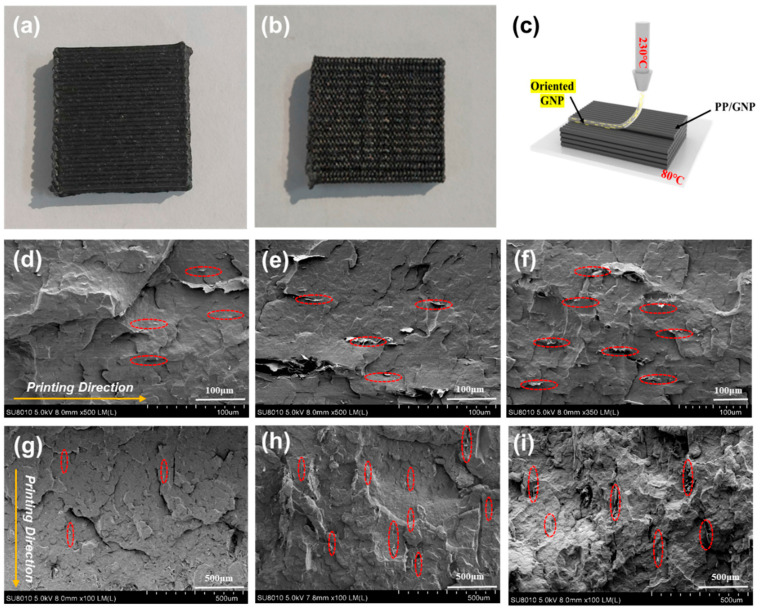
FDM printed parts of FP (**a**) and VP sample (**b**); GNP orientation behavior of iPP/GNP composites during FDM printing process (**c**)**;** SEM results of various FDM printed parts (**d**) FP1, (**e**) FP3, (**f**) FP5, (**g**) VP1, (**h**) VP3, (**i**) VP5. The red circle represents the aligned GNP particles within iPP matrix.

The dispersion and orientation of graphene within the matrix are crucial to the thermal conductivity of the 3D-printed parts. Uniformly dispersed GNPs would mitigate thermal resistance, thereby facilitating heat transfer throughout the composite material. Graphene, with its two-dimensional sheet-like structure, offers pronounced thermal conductivity anisotropy; that is, it conducts heat more efficiently along its planes than perpendicularly. Consequently, regulating GNPs along the printing direction can substantially enhance the thermal conductive efficiency, particularly when aligned with the direction of heat flow. Accordingly, the cross-sectional morphologies of the FP and VP printed parts were evaluated. Figure 4a,b present the overall digital image of the as-fabricated FP and VP parts, respectively. It can be seen that both of the parts displayed a good appearance with distinct boundaries between adjacent layers through consistent spacing and a high degree of dimensional accuracy. No voids were observed in the cross-section of FP and VP printed parts, indicating good interlayer bonding. Good interfacial adhesion was observed between GNPs and iPP matrix at a low GNP content, while slight GNP agglomeration was detected at a high GNP content, which may be detrimental to the mechanical properties of printed parts. During the FDM process, the anisotropic fillers would align following the printing direction, as illustrated in Figure 4c. Consequently, this mechanism allows control over the orientation of GNPs with the iPP matrix. Regardless of the FP or VP sample, the GNPs were aligned along the printing direction, as shown in Figure 4d–i. In addition, as the GNP content increased, more oriented graphene could be observed in the printing direction, which was beneficial to the improvement of the thermal conductivity of composites. Since the in-plane thermal conductivity of the FP sample depended on the thermal conductivity of the GNPs in the vertical direction, it can be expected that the thermal conductivity of the FP sample will be inferior to that of the VP sample.

### 3.4. Thermal Behavior of FDM Printed iPP/GNP Composites

**Figure 5:**
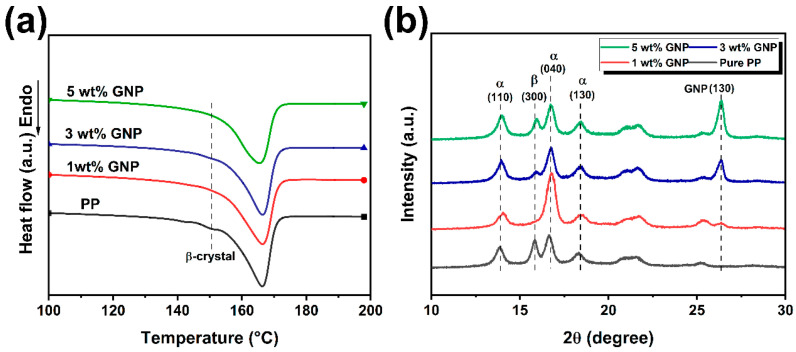
(**a**) DSC curves and (**b**) XRD pattern for iPP/GNP FDM printed parts with various GNP loadings.

XRD and DSC tests were employed to characterize the crystal structure of the printed composites. The XRD patterns of the printed composites of various GNPs are presented in Figure 5a. It was seen that α-crystal reflection peaks with 2θ values of approximately 14°, 16°, 16.8°, and 18.6°, corresponding to (110), (040), and (130) reflections, appeared in all four samples [44]. In addition, a peak at 2θ = 16° accounted for the (300) plane of β-crystals was also observed. The diffraction peak at 2θ = 26.4° is the characteristic peak of the (200) plane for GNPs, and its intensity increased as the GNPs content was raised. Notably, the diffraction peak position of the composites remained consistent with that of the pure iPP samples, suggesting that the addition of GNP did not alter the crystal structure of the iPP. It can be found that the addition of GNP significantly influenced the crystalline behavior of iPP crystal form. Pure IPP exhibited a certain amount of α and β-crystals. At a GNP content of 1 wt%, β crystals virtually disappeared, with a domination formation of α-crystals. Upon further increase of GNP content, β-crystals reemerged with their content increased with increasing GNP content. It was conceivable that the pronounced shear field during FDM processing can promote the alignment of GNP particles and iPP molecular chains along the printing direction, generating two primary kinds of nucleating sites in the FDM printed composite parts. One was the heterogeneous nucleation sites offered by the aligned GNP particles, with a large specific surface area. The iPP molecular chain could efficiently interact well with the GNP surface, thereby reducing the nucleation barrier and favoring the growth of α-crystals. In addition, other homogeneous nucleation sites also existed in the composite parts, manifested as row nuclei and induced the formation of β-crystals, which was due to the synergistic effect of a strong shear field at the FDM extrusion state and the large temperature gradient during the deposition stage. As the GNP content increases, the stability of core formation can be improved. It should be noted that the relaxation time of the oriented polymer chains was extended due to the confining effect of GNP, making row nuclei develop into “shish” entities [45]. Given this, the introduction of GNP predominantly favored α-crystals within the printed parts, which was attributed to the exceptional anisotropic nucleation property of GNP. Simultaneously, as GNP content escalated, the formation of a network structure facilitated the stability of row nucleation and acted as an effective “shish”, further triggering the emergence of β-crystals. Figure 5b illustrates the DSC test results of neat iPP and its GNP composite printed parts. Strong α and weak β endothermic peaks are shown in neat iPP, while no β-crystals melting peaks were observed for composite parts. It is well known that α-crystals are a thermodynamically stable phase, while β-crystals are a thermodynamically metastable phase. During the DSC test process, it was easy for β-crystals to undergo β/α crystal transition and exothermic when heating up. This would partially offset the endothermic signal associated with the β-crystals, leading to the disappearance of the endothermic peak [46]. Additionally, the introduction of GNP improved the crystallinity of printed parts to a certain extent, but as the GNP content increased, the crystallinity decreased slightly, which may be attributed to the restriction effect of high-content GNP on the movement of iPP molecular chains.

### 3.5. Thermal Conductivity of FDM Printed Samples

**Figure 6:**
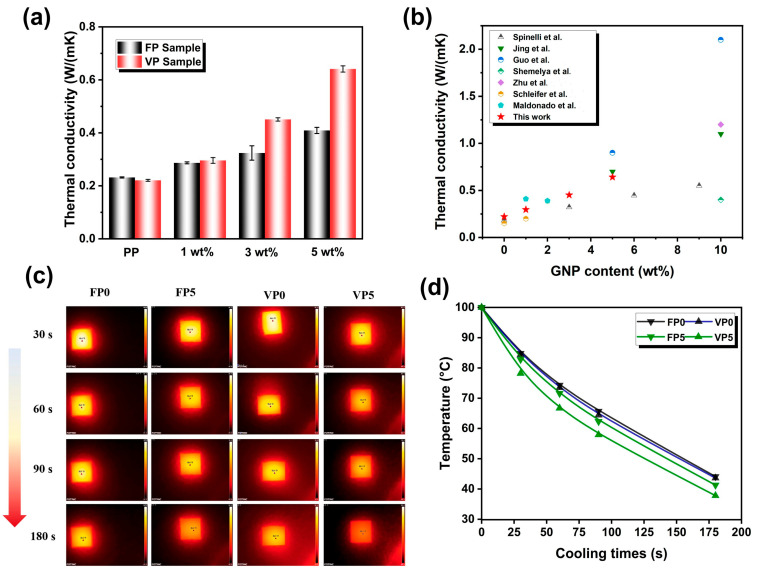
(**a**) Thermal conductivity of FP and VP printed parts; (**b**) Comparison of thermal conductivity of the as-printed composites with those of previously reported works [34,35,47,48,49,50,51]. (**c**) Thermal imaging test results of different printed parts for FP0, FP5, VP0, and VP5 samples; (**d**) Temperature changes curve of various FDM printed parts.

The anisotropic thermal conductivity of graphene nanoplatelets (GNP) can induce a corresponding anisotropy in the thermal conductivity of a polymer matrix. To evaluate the effect of the 3D printing construction method on the thermal conductivity of composites, the thermal conductivity through the thickness direction of the FDM printed parts was studied, as shown in Figure 6. As presented in Figure 6a, the inherent low thermal conductivity of neat iPP, coupled with the lack of oriented high thermal conductivity functional fillers in the matrix, resulted in a low thermal conductivity of 0.23 W/m · K and 0.22 W/m · K for FP0 and VP0, respectively. Conversely, the FDM-printed iPP/GNP composites exhibited a clear increase in thermal conductivity across both printing directions with the addition of GNP, escalating from 0.29 W/m · K of FP1 to 0.42 W/m · K of FP5 and from 0.30 W/m · K of VP1 to 0.64 W/m · K of VP5. Remarkably, the thermal conductivity of the VP samples surpassed that of the FP samples, a distinction particularly pronounced in the VP3 (0.46 W/m · K) and VP5 (0.64 W/m · K) samples, yielding an increase of approximately 109% for VP3 and 191% for VP5 compared to that of their FP samples, respectively. Notably, the VP5 sample achieved a thermal conductivity value of 0.67 W/m · K, which is comparable to the recently reported thermal performance of graphene composites fabricated by using 3D printing, as presented in Figure 6b. Thermal imaging was conducted to visually investigate the thermal conductivity differences of the FDM printed parts to observe the thermal diffusion behavior during heating and cooling cycles, as illustrated in Figure 6b. It was apparent that the tested sample temperatures declined at different rates, with VP5 and FP5 samples exhibiting a more rapid temperature drop over time compared to VP0 and FP0 samples during the heat dissipation process. The FP0 and VP0 samples presented the slowest temperature response, hindered by their inadequate heat dissipation capabilities, leading to the formation of hot spots at the center of the tested samples. As shown in Figure 6c, the temperature of FP samples dropped from 100 °C to 41.3 °C within 180 s, while the VP5 samples decreased from 100 °C to 37.8 °C, indicating enhanced heat flux diffusion and conduction efficiency of VP5. Overall, the infrared thermal images and corresponding temperature curves illustrated that the heat dissipation capacity among the printed parts arranged as VP5 > FP5 > VP ≈ FP.

**Figure 7:**
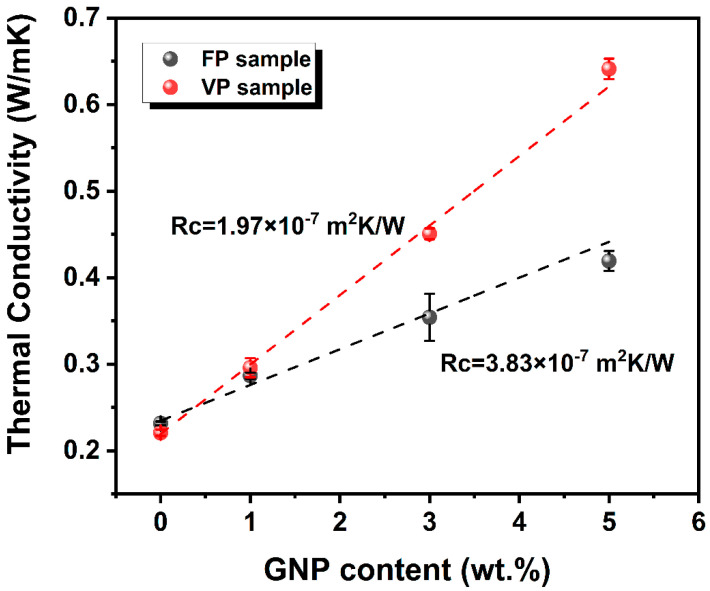
Extracted thermal interface resistance values of the FP and VP parts of nanocomposites based on the EMT model.

To evaluate the impact of 3D printing construction methods on the phonon transmission, the interface thermal resistance of VP and FP printed parts was calculated by utilizing the effective medium theory (EMT) [52,53]. In this context, the thermal conductivity within the plane of the composites can be mathematically represented as the following equations:

$$k_{c}=k_{m}\frac{3+V_{f}(\beta_{⊥}+\beta_{∥})}{3-V_{f}\beta_{⊥}}$$

$$\beta_{⊥}=\frac{2[d\left(k_{f}-k_{m}\right)-2R_{1}k_{f}k_{m}]}{d\left(k_{f}+k_{m}\right)+2R_{1}k_{f}k_{m}}$$

$$\beta_{∥}=\frac{L\left(k_{f}-k_{m}\right)-2R_{1}k_{f}k_{m}}{Lk_{m}+2R_{1}k_{f}k_{m}}$$

where $k_{c}$, $k_{m}$, and $k_{f}$ are the effective thermal conductivity of the polymer composites, neat iPP and the GNP filler, respectively. $V_{f}$ are the volume fraction of GNP within the composites, and $R_{I}$ represents the interfacial thermal resistance between GNP and the polymer matrix. Additionally, L and d are the lateral size and thickness of GNP, respectively. In this study, iPP was utilized as the matrix with a thermal conductivity of 0.22 W/m · K. The thermal conductivity of the GNP filler was established at 2000 W/m · K, with a lateral size (L) of 10 μm and a thickness (d) of 15 nm. Based on the above equations and the experimental measurement of $k_{c}$, the fitting situation of $R_{I}$ values of the FP and VP printed samples are shown in Figure 7. It can be seen that the $R_{I}$ for the VP sample was calculated to be 1.97 × 10^−7^ m^2^ K/W, which was lower than that of the FP sample at 3.83 × 10^−7^ m^2^ K/W. This distinction implied that the VP samples benefited from a more thermally efficient interface, which could be well attributed to the alignment of GNP, which was more parallel to the heat conduction path, minimizing the scattering and disruption of heat flow across the interface.

**Figure 8:**
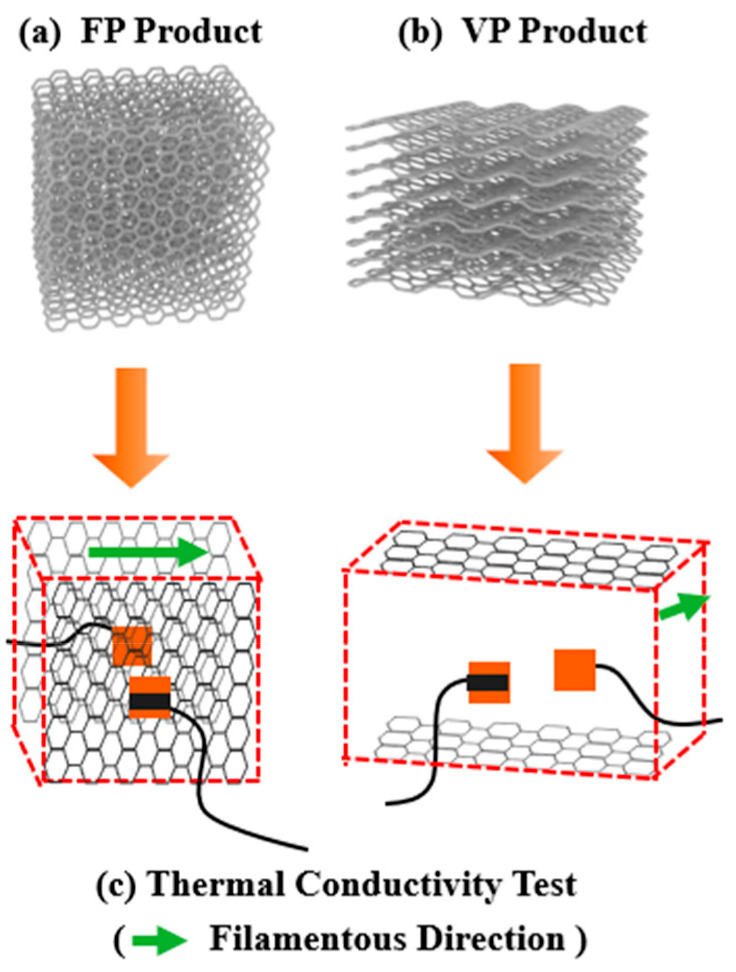
Schematic diagram of thermal conduction path of FDM printed parts: (**a**) FP sample, (**b**) VP sample. (**c**) The constructed thermal pathway within FP and VP samples.

The mechanism of the difference in thermal conductivity between FP and VP of FDM printed parts is shown in Figure 8. In the situation of the FP sample, the heat flow produced by the heating device encountered blocks at each layer interface due to the vertical orientation of GNP with respect to the heat flow direction. More importantly, the heat flow conduction necessitated bridging the gap between two adjacent filaments within the printed parts, generating a significant interface thermal resistance and thus serving as a barrier to hinder the efficient conduction of phonons. This structure arrangement disrupted the thermal path continuity, leading to higher interfacial thermal resistance and lower overall thermal conductivity. However, as for the VP sample, the GNPs were aligned along the printing direction, which was also the heat flow direction, providing a continuous pathway for heat transfer and minimizing interruptions at printed layer interfaces, thus facilitating an efficient heat transfer. In this case, the superior in-plane thermal conductivity of GNP could be fully exploited, allowing for a more direct and shortened heat transfer path, as presented by the red arrow in Figure 8, thereby enhancing the overall thermal conductivity of VP samples.

### 3.6. Mechanical Properties of the FDM Printed Samples

**Figure 9:**
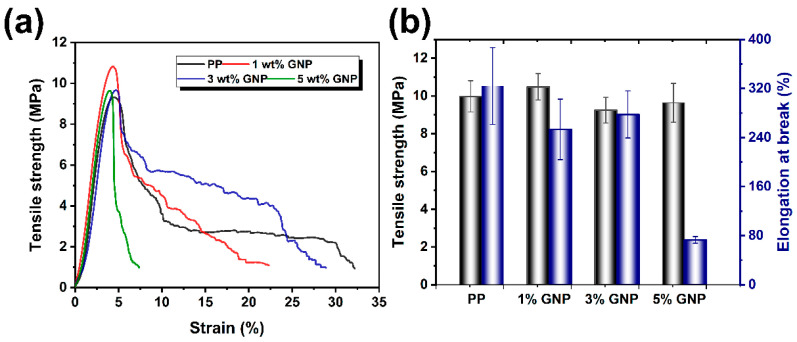
(**a**) Stress-strain curves and (**b**) tensile strength and elongation at break for printed samples of iPP/GNP composite with various GNP loadings.

Figure 9 presents the mechanical properties of FDM printed samples for neat iPP and iPP/GNP composites with various GNP loadings. Notably, the incorporation of 1 wt% GNP into the iPP matrix resulted in a slight increase in tensile strength by 0.51 MPa compared with the neat iPP. This is in line with XRD results, where a predominance of α-crystals at 1 wt% GNP contributed to the enhanced mechanical properties. However, with the further increase of the GNP content, a notable decline in the tensile strength was observed. This reduction could be well explained by the agglomeration of GNP at high content, which indeed acted as a stress concentration point and faded the effectiveness of load transfer. The XRD results showed that a high GNP content was beneficial to the formation of β-crystals, thus improving the mechanical performance of the printed parts. However, this would be counteracted by the adverse effect of GNP agglomeration. Especially when the content of GNP increased to 5 wt%, the elongation at break reduced sharply from 323.84% of pure iPP to 72.9%. This significant loss of ductility can be correlated with the GNP agglomeration behavior, as also presented in the SEM images, which compromised the interfacial adhesion between GNP and iPP matrix, leading to premature failure and reduced deformability subjected to tensile stress.

## 4. Conclusions

In conclusion, by adopting the strategy that the orientation of fillers can significantly enhance the thermal conductivity of composites, we have successfully utilized FDM 3D printing technology to tune the orderly alignment of GNPs within a universal iPP matrix, thereby fabricating iPP/GNP thermal conductive composites with superior properties. Rheological analysis indicated that the inclusion of GNPs not only enhanced the viscosity of the composite system but also promoted shear thinning behavior, facilitating a smooth extrusion through the FDM printer nozzle. It was estimated that the shear stress encountered by the composites within the nozzle could reach about 0.73 MPa, assisting in the orientation of the GNPs. Polyflow simulations further demonstrated that GNP addition resulted in an increased pressure drop and shear rate during FDM extrusion, with the shear rate stabilizing around 200 s^−1^. This condition favored the alignment of GNPs along the printing direction, as supported by SEM observations. Exploiting the FDM-induced orientation of GNPs, the VP printed components exhibited a thermal conductivity of 0.64 W/m · K at a 5 wt% GNP loading, substantially surpassing the 0.42 W/m·K thermal conductivity of FP samples. Remarkably, the interfacial thermal resistance of VP printed parts was reduced to 1.97 × 10^−7^ m^2^ · K/W, significantly lower than the 3.83 × 10^−7^ m^2^K/W observed in FP samples, highlighting the effectiveness of filler orientation in boosting thermal conductivity. This investigation not only highlights the potential of oriented GNP structures in enhancing the thermal management of PP-based composites but also sets a new benchmark for the development of complex-structured thermally conductive materials, particularly in applications such as LED lighting, where efficient heat dissipation is crucial. In the future, by altering the flow field during the FDM process, such as the printing speed, and employing hybrid fillers, like integrating GNP with MWCNT or BN, to diversify the construction of thermal conduction pathways, the thermal performance of printed parts can be further enhanced.

## Figures and Tables

**Table 1:** FDM printing parameters.

Filament diameter (mm)	1.75
Nozzle diameter (mm)	0.40
Printing temperature (°C)	230
Platform temperature (°C)	80
Layer thickness (mm)	0.2
Filling rate (%)	100
Filling angle (°)	0–0
Printing speed (mm/s)	10

## Data Availability

Data are contained within the article.
